# Supplementary material for: Are carbon emissions trading and green financial instruments synergistic? -Comprehensive quantitative research based on content analysis
Source: PLoS One. 2024 Mar 7;19(3):e0298601. doi: 10.1371/journal.pone.0298601 (PMC10919601; doi:10.1371/journal.pone.0298601)
Supplement: S1 Data — (ZIP) [file pone.0298601.s001.zip › Data/地级市Z_F工作报告.zip!/╣π╢1⁄2/╒┐╜¡╩╨/└┤╫╘╣1⁄2╓┌║┼í╛┬φ┐╦╩2╛▌═°í┐_2008.docx]

各位代表：

　　我代表市人民政府向大会作政府工作报告，请予审议，并请市政协委员和其他列席的同志提出意见。

　　2007年工作回顾

　　2007年是新一届市政府推进科学发展、促进社会和谐取得明显成效的一年。一年来，市政府在中共湛江市委的正确领导下，在市人大及其常委会和市政协的监督支持下，认真贯彻党的十七大、省第十次党代会和市第九次党代会精神，全面贯彻落实科学发展观，深入实施“工业立市、以港兴市”发展战略，狠抓各项工作落实，圆满完成了市十二届人大一次会议确定的各项目标任务。

　　经济综合实力明显增强。全市实现生产总值892.6亿元，增长13%，增速创12年来新高。其中，第一产业增加值176.7亿元，增长5%；第二产业增加值423.1亿元，增长14%；第三产业增加值292.8亿元，增长16.3%。人均生产总值13217元，增长12%，比2000年翻一番。单位生产总值能耗下降2.7%。全市地方财政一般预算收入38.2亿元，增长25.8%，增长率高于全省东西两翼平均增长水平1.23个百分点；来源于湛江的财政总收入190亿元，增长28.8%。城市综合实力位居全国百强城市之列。

　　产业结构继续优化。全市三次产业比例为19.8∶47.4∶32.8，其中第二产业比重提高1.3个百分点，第三产业比重提高0.5个百分点，第一产业比重下降1.8个百分点，首次降到20%以下；轻重工业增加值比例为32.5∶67.5，重工业比重高于全省6.5个百分点，产业适度重型化趋势不断增强；第三产业增加值增幅首次超过第二产业，高出2.3个百分点。

　　工业经济跨上新台阶。全市工业总产值首次突破1000亿元大关，达到1121.3亿元，增长17.1%，标志着我市工业进入新的发展阶段。工业企业经济效益综合指数270%，居全省前列。工业产品新增“中国名牌”8个，新获中国驰名商标1件、广东省著名商标10件。工业对经济增长的贡献率46.1%，在三次产业中对全市经济增长的贡献最大。

　　现代农业和新农村建设扎实推进。成功抗御两百年一遇特大洪水灾害，农业生产保持稳定增长。实现农业总产值293.5亿元，增长5%。粮食生产圆满完成省下达的任务；水产品产量97万吨，居全省首位；产糖量130万吨，再创历史新高；畜牧产业规模化水平超过30%。落实粮食最低收购保护价和大宗农产品减免收费，整顿农产品市场价格秩序，减轻农民负担，增加农民收入。全市投入乡村道路、农田水利和农村居住环境建设改造等资金41.7亿元，增长21%；31项城乡水利防灾减灾工程已有28项动工建设，累计完成投资8.44亿元。新建农村硬底化公路1300公里。动工建设硇洲国家级中心渔港。完成造林24.2万亩，森林覆盖率提高到25.4%。农业产业化取得新发展，新增省级农业龙头企业4家、省级示范性农民专业合作社12个。31个农海产品获国家、省名牌称号或质量认证。新农村建设呈现以创建生态文明区（片、带）为载体、连点成片集群发展的新格局。全市完成1125条村庄规划编制，建成生态文明村300条，其中11条受省表彰。

　　县域经济发展成为新亮点。县域完成生产总值385亿元，增长12.6%。县（市）工业经济提速增效，规模以上工业实现总产值220.6亿元，增长26.2%。廉江市成为我市第一个生产总值和工业总产值双超百亿元的县（市）。县域财政一般预算收入增长30.9%，其中吴川、雷州、廉江三市超过全省平均增长水平，各县（市）财政综合增长率都超过省财政激励机制奖的指标。

　　基础设施和城市功能不断完善。全市完成固定资产投资242.8亿元，增长17.4%，相当于2003年投资额的两倍；其中重点项目建设完成投资58.5亿元。中石油98万立方米燃料油库扩建、湛江发电厂脱硫、霞山污水处理厂首期等7个项目建成；黎湛铁路复线河唇至湛江段即将竣工；龙腾物流500万吨球团、湛江港30万吨级航道疏浚等16个项目动工建设。编制完成东海岛、开发区东海岛新区等2个分区规划和5个控制性详细规划，全面完成19个中心镇总体规划编制和评审工作。全市土地入库3237.5亩。完成一批城市骨干道路建设，改造城区70条小街小巷。动工建设赤坎污水处理厂二期工程。合理调整市区公交线路网站，新建一批市区公交候车亭，首批油改气环保型出租车投入营运。建成绿塘河湿地公园、中澳友谊花园、万象棕榈园和一批小游园、小绿地，新增城市绿地面积106.2万平方米，城市建成区绿地率40.6%，绿化覆盖率45.7%，居全国重点城市第四位；人均公共绿地面积11.6平方米，城市环境综合质量连续四年排名国家环保重点城市前五位。被评为全国10大休闲城市之一。省级湖光红树林湿地公园申报获得批准。徐闻珊瑚礁保护区和雷州湾中华白海豚保护区分别被批准为国家级自然保护区和国家一级保护动物自然保护区。麻章区被评为全省林业生态县。全面实施城乡清洁工程，全市动员、全民动手整治“脏、乱、差”现象，城乡生态环境面貌焕然一新。

　　第三产业增长势头良好。港口物流、交通运输、商贸流通、金融保险、旅游、房地产等重点服务行业快速发展，消费需求不断扩大，消费市场购销两旺。全年社会消费品零售总额382.2亿元，增长19.5%，创12年来新高。全市港口货物吞吐量达到9165万吨，增长12.1%，为奋进亿吨港口奠定了坚实基础。多家高星级酒店动工，房地产开发投资突破30亿元，创历史新高。接待游客769万人次，增长12.6%；旅游业总收入41.3亿元，增长11.4%。成功举办中国（广东）国际对虾节。

　　外向型经济成效显著。全年外贸进出口25.75亿美元，增长19.9%，其中外贸出口14.6亿美元，增长19.2%。水产业顺利通过美国、俄罗斯、韩国等国家有关部门的检查，国联水产有限公司成为我国首家获美国解除自动扣检限制的水产出口企业。对外招商引资取得新突破，实际吸收外资1.5亿美元，增长1.86倍，扭转了多年来低位徘徊的被动局面。

　　体制改革迈出新步伐。国有企业改革取得突破性进展，湛江港集团有限公司通过引进战略投资者招商局集团，成功改制为股份有限公司，新体制为湛江港集团加快发展注入了强大活力；市直14家国有企业平稳退出市场，盘活了存量资产；初步建立国有资本经营预算制度；国有粮食体制改革取得重大进展，政策性关闭企业123家，新组建企业运转良好；全面完成县（市）供电和价格体制改革，为群众和企业减轻电费负担3.2亿元。金融体制改革稳步推进，金融、证券、保险业务稳健快速发展，银行业机构实现税前利润9.4亿元，增长113%，市商业银行税前利润首次突破2亿元，增长66.7%；启动农村信用社改革试点，5家停业整顿城市信用社如期进入撤销程序，获得省奖励；发展利用资本市场步伐加快,确定5家拟上市公司和5家上市后备企业。财政支出管理改革进一步深化，市直预算单位全部实行集中支付管理并扩展到县（市、区），对145个市级财政支出项目开展绩效评价。非公有制经济发展迅速，全市民营企业8705户，增长25%，个体工商户11.6万户，增长21%；民营企业增加值386.3亿元，增长15.5%。

　　各项社会事业协调发展。建立区域性特色产业基地1个、技术创新试点专业镇3个；组织实施国家、省、市级科技计划项目223项、省部产学研合作项目4项，新增省级高新技术企业12家，全市高新技术产品总产值163.8亿元，增长20%；组建省级工程中心2家、市级工程中心和农业技术创新中心3家、技术研发机构12家，取得科研成果66项，专利申请量642项、授权量482项，分别增长10.1%和44.3%；科技进步对工业经济增长贡献率提高到47%。投资3.69亿元完成农村中小学校舍C、D级危房改造任务，拆除危房54万平方米，新建校舍47万平方米，维修校舍29万平方米；免收农村和城市低保家庭义务教育书杂费，直接减轻群众负担4.8亿元；高中阶段教育和职业技术教育取得新成效，有2所中学通过国家级示范性高中验收，高中阶段毛入学率比上年提高6.6个百分点，中等职业技术和技工学校招生增长15%。成立市非物质文化遗产保护中心；荣获全国第14届艺术“群星奖”创作奖；建成市广播电视中心。完善城乡三级医疗预防保健网络，新增城市社区卫生服务机构9个，扩大平价医疗服务，调整3900多个医疗服务项目，收费综合下降5.3%，减轻群众医疗负担；重大动物疫病免疫密度100%，有效预防禽流感等疫情；10条村被评为“广东省卫生村”。竞技体育成绩喜人，我市运动员荣获世界大赛金牌3枚、亚洲比赛金牌2枚、全国比赛金牌15枚；参加第六届全国城运会赛事项目和人数实现历史性突破；成功举办首届国际龙舟邀请赛。圆满完成人口与计生各项目标任务，出生人口性别比下降1.2点。国防动员体系不断完善，民兵预备役建设不断加强，第三次荣获全国双拥模范城称号。建立健全全市公共应急处置机构和雷州半岛气象、水文、地质等灾害监测预警系统。率先在全省完成小城镇档案工作。统计、城调、供销社、侨务、外事、港澳、对台、人防、气象、地震、民族、宗教、地方志、残疾人事业等各项工作取得新的成绩。

　　人民生活水平不断提高。市区城镇居民人均可支配收入11389元，增长9%；全市农村居民人均纯收入4980元，增长7.8%。全年新增就业岗位5.2万个，完成年度任务的130%；下岗失业人员实现再就业1.8万人，完成年度任务的120%，其中“4050”人员再就业6900人，完成年度任务的115%；60%的社区基本实现充分就业。组织农村青年和退役士兵免费技能培训4万人，转移农村劳动力就业17万人。安置残疾人就业3000名，残疾人就业率85%以上。进一步完善最低生活保障制度，城乡15.2万生活困难群众列入最低生活保障，3.37万低收入群众获得物价补贴。全市企业养老保险增加6万人、失业保险增加2万人、工伤保险增加3.5万人。启动职工生育保险。开展孤儿关爱行动，全市8611名社会散居孤儿得到结对帮扶。实施城镇居民医疗保险国家试点城市工作，首批参保22.2万人，参保率达68.5%；提高新型农村合作医疗补偿标准，参合人数399万人，参合率达77.4%。完善城镇住房保障制度，建成廉租房26474平方米，部分住房困难群众居住条件得到改善。大力推进农村安居工程建设，投入农村困难群众危房改造资金9.82亿元，开工建房25980户，已竣工20362户，在建5618户，解决了6.87万困难群众的住房难；扶持在去年特大洪灾中房屋全倒的3671户村民兴建新房，今年春节前全部搬进新居。新建村级敬老院125间，解决2500名五保老人住房困难。

　　民主政治和文明法治建设取得新成效。全年办理省市人大代表议案及建议208件、满意率95.7%；办理政协委员提案193件、满意率96.9%。全面开展以“四服务两促进”为主题的机关效能建设活动，大力推行行政问责制，建成电子行政审批和电子监察系统，496项行政审批事项纳入监管，项目报建累计审批时限从229个工作日缩减到63个，平均每项审批事项的办结时限由21个工作日压减到7.5个。政务公开工作进一步扩大和规范，市直部门全部公开作出服务承诺，麻章区被评为全国政务公开工作先进单位和示范点。开展对执行“十项纪律”的督查，首次在媒体公开审计结果。认真开展专项治理工作，坚决纠正损害群众利益的不正之风。积极调控结构性价格上涨，物价指数低于全国、全省平均水平。启动实施“五五”普法规划，1.62万人次得到法律援助。落实安全生产责任制，有效遏制和防范重特大安全事故发生。开展产品质量和食品安全专项整治行动，全面完成整治目标任务。实施领导干部基层大接访和包案制度，市、县两级政府处理信访大要案1340多宗，全市信访总量下降38.6%。推进公安机关“三基”工程和城乡治安防控体系建设，促进治安管理和防范社会化，继续严厉打击“两抢一盗”等严重违法犯罪和城乡黑恶势力，坚决扫除黄赌毒等社会丑恶现象，城乡平安和谐，社会保持稳定，刑事案件立案率下降8.3%，连续四年被省评为“社会治安综合治理工作优秀市”。深入开展打击走私综合治理，在全省考核中取得满分的好成绩。群众性精神文明创建活动深入开展，创建安全文明小区6036个、平安示范村10个、省和市安全文明校园40所；全市11个文明单位、4个文明社区、4个文明镇受省表彰；32个城市社区被评为全省“六好”平安和谐社区。

　　过去一年取得的成就，是在中共湛江市委正确领导下全市人民团结奋斗的结果，凝聚着社会各界的辛劳和智慧。在此，我代表市人民政府，向各级人大代表和政协委员，向全市广大工人、农民、知识分子、干部，向驻湛人民解放军、武警官兵、人民警察和各民主党派、人民团体、社会各界人士，向所有关心和支持湛江发展的港澳台同胞、海外侨胞和国际友人，表示崇高的敬意和衷心的感谢！

　　在充分肯定成绩的同时，也要清醒认识到，我们的工作与新时期科学发展的要求，与全市人民群众的期待还有差距，经济社会发展还存在不少困难和问题。突出的是：经济结构不够合理，支柱产业对经济增长的支撑力度不够强，第二产业占生产总值的比重未达全省平均水平，第三产业尤其是现代服务业发展不快，高新技术产业比重不高；城乡、区域发展不够协调，县域经济基础薄弱，对农业和农村经济带动力不强，农村集体经济发展滞后，农民增收不稳定；经济外向度不高，外贸出口和实际利用外资总量偏小，龙头型、基地型外商投资项目不多；城乡规划建设和管理亟待加强，旧城区和城中村改造推进缓慢，违法违规建设问题比较突出；经济发展方式还比较粗放，节能降耗和治污减排监管力度不够；社会事业发展水平不够平衡，就业、社保、教育、医疗、住房等方面问题依然较多，部分低收入群众生活还比较困难；政府职能和机关作风仍未完全适应新时期、新形势、新任务的要求，运用市场经济手段破解难题、推动创新的能力不强，有的部门和领导抓主动服务、抓工作落实不到位。对此，我们一定要高度重视，以更强的紧迫感和责任感，采取更有力的措施认真解决，努力把各项工作做得更好。

　　2008年工作安排

　　2008年是全面贯彻落实党的十七大作出的战略部署的第一年，是实施“十一五”规划承上启下的一年，做好今年的工作意义重大。我们要紧紧围绕发展与和谐两大主题，按照稳中求快、好字优先、改革开放、以人为本的原则，以及抓落实、优发展、重民生、促和谐的工作方针，进一步推动经济社会又好又快发展。

　　今年市政府工作的总体要求是：全面贯彻党的十七大精神，深入贯彻落实科学发展观，继续解放思想，坚持改革开放，坚持以经济建设为中心，加快工业化进程，强化港口龙头带动作用，统筹城乡发展，建设生态文明，着力改善民生，促进社会和谐，建设城乡协调、生态文明的科学发展试点市，努力实现湛江经济社会全面振兴。

　　今年我市经济社会发展的主要预期目标是：全市生产总值增长11%，确保超1000亿元；单位生产总值能耗下降2.75%，二氧化硫和化学需氧量分别削减2%和3%；地方财政一般预算收入增长11%；全社会固定资产投资增长15%；社会消费品零售总额增长16%；居民消费价格涨幅控制在4%左右；外贸出口总额增长10%，实际吸收外资增长15%；城镇居民人均可支配收入增长8%，农村居民人均纯收入增长6%；城镇登记失业率控制在3.5%以内；人口自然增长率控制在6.8‰以下。

　　今年的主要任务和工作重点是：

　　一、坚持工业主导型增长格局，推进现代产业体系建设

　　加快发展工业支柱产业。进一步强化工业主导地位，加快工业化进程。一是以推进重点项目建设加快工业发展。加快建设龙腾物流500万吨球团、70万吨木浆、东兴炼油配套完善、澳里油电厂油改煤、环球钢轮扩建、广东冠豪特种防伪纸、尧丰包装印刷等重点工业项目；全力支持中石化、中石油、中海油在湛企业增资扩产；积极推动重化能源项目向县（市）布局；继续扎实做好湛江钢铁项目前期工作。抓好国家和省规划在湛建设的重点项目跟踪落实，力争更多项目开工。二是以推进资源要素整合加快工业发展。建立深度加工企业优先发展和资源加工经济利益共享机制，引导资源和生产要素向优势企业集中，推动农海产品加工及家用电器、制糖、造纸等产业实施品牌、资产重组，形成关联度强、配套成本低、竞争优势明显的同业集聚型和龙头带动型产业基地。三是以推进企业集团化加快工业发展。着力培育一批主业突出、市场竞争力强、带动全市工业跨越发展的龙头企业集团，市级重点培育一批年产值10亿元以上企业集团，县级重点培育年产值1亿元以上企业集团，以大企业带动工业大发展。评选公布全市工业企业50强，继续表彰工业发展先进县（市、区）、乡镇。四是以推进园区建设加快工业发展。加快湛江经济技术开发区东海岛新区和临港工业园区基础设施建设，抓紧上马一批上规模、有影响的临港工业项目。坚持以园区为载体，以民营为主体，发展壮大支撑县域经济发展的工业支柱产业。重点支持佛山顺德（廉江）、深圳龙岗（吴川）省级产业转移工业园完善基础设施，承接更多项目落户；支持其他县（市）和中心镇、专业镇建立产业转移工业园和兴办工业集中发展区，力争年内一批产业转移项目动工或建成投产，形成县域经济新的增长点。

　　加快发展港口物流业。抓住湛江港集团与招商局集团成功实行战略合作的机遇，加快建设临港物流园区和保税物流中心，努力拓展综合批发、期货交割、保税中转等物流业态，完善港口大型专业市场，推进实施中港印1000万吨配煤中心、中航油150万立方米储油等港口物流项目，建设适应石油、矿石、煤炭等大宗能源、原材料货物集散地，适应以大宗散货为主体的外贸货物调运、仓储、加工、分拨、转口贸易的物流基地，确保全年港口货物吞吐量突破亿吨大关。

　　加快发展现代服务业。坚持把服务业作为建设现代产业体系的重要抓手，进一步加快发展速度，提高产业比重。积极发展商务、保险、信息、会展等现代服务业，促进传统服务业向特色经营、树立品牌的新型业态提升，引导社区服务业向公共需求大的领域发展。改善优化金融生态环境，支持金融业打造粤西和北部湾区域金融中心。启动海滨大道海湾大桥至赤坎旧大天然酒店路段的改造和开发，重点建设一批高星级酒店和购物、商住、旅游消费项目，倾力打造城市商务中心区。推进广州湾时代广场、渔人码头等商贸流通重点项目建设。加快建设粮食、农资、花卉园艺等专业市场和大型农产品、水产品批发中心。引入更多有实力的房地产企业和知名品牌，建立高中低档结构合理的住房供应体系，推动房地产业健康持续发展。推进“万村千乡”市场工程和农产品连锁经营试点，积极发展农村服务业。继续举办广东湛江国际对虾节。着力发展旅游休闲业，坚持以湖光岩世界地质公园和东海岛旅游度假区为龙头，有效整合蓝色滨海、绿色生态、红土文化和特色产品等优势资源，全力打造“海滨港口园林城市、度假休闲胜地、海鲜美食天堂”的旅游城市品牌。完善公园和景区服务设施，建设一批旅游休闲服务项目。充分利用新农村建设成果，加快开发特色乡村旅游，形成生态旅游新亮点。加强与琼北、两广十市和泛珠三角旅游区域协作，共建无障碍旅游市场。

　　加快发展高新技术产业。坚持以自主创新促进产业优化升级，引导创新要素向企业集聚，大力支持企业建立研发中心，推动技术创新、管理创新、产品创新，促进经济发展方式从生产要素驱动型向技术创新驱动型转变。建立集智攻关协同机制，推动重要科技专项研发快出成果、早出产品，带动相关新兴产业发展。推进实施一批电子信息、先进制造、生物医药、新能源、新材料等高新技术项目，鼓励更多企业转向生产技术含量高、附加值高的产品。实施20个促进产业升级、实现技术跨越的重点技改项目。积极实施品牌带动战略，鼓励企业获得更多品牌和国内、国际认证。全年高新技术产业总产值增长15%以上。

　　二、统筹城乡发展，推进社会主义新农村建设

　　扎实推进现代农业建设。充分利用气候区划成果，进一步优化农产品区域布局，推动农业品种资源开发利用，重点建设糖蔗、红橙、菠萝、香蕉、北运菜、花卉、剑麻、桉树、珍珠、对虾十大优势农产品产业带，壮大农业主导产业。着力培育城郊型农业，重点支持麻章花卉产业带和湛江南国花卉科技园建设。加强市、县农产品质检机构建设，创办更多农产品标准化示范基地，培育更多无公害产品、绿色食品、有机食品和名牌产品，做强做大品牌农业。强化农业招商引资，加快海峡两岸农业合作试验区建设步伐。推进农业产业化经营，支持农业龙头企业加大科技投入，提升农海产品精深加工水平，壮大延伸对虾、珍珠、果菜、畜牧、热作和林木六大特色产业链。坚持依法自愿有偿原则，健全土地承包经营权流转制度，发展多种形式的适度规模经营。鼓励农民围绕产前、产中、产后等环节开展多元化、多形式的合作，支持发展农民专业合作经济组织，培育农村经纪人，在有条件的农村大力推行集体经济股份制改革，提高农业生产和农民组织化程度。

　　扎实推进农业基础设施建设。注重从战略和全局高度研究解决淡水对全市经济社会发展的制约问题，坚持开源节流，双向治本，着眼长远，毫不松懈抓好治旱防旱工作。实施雷州半岛扩大治旱、雷州青年运河灌区改造、中小型水库除险加固和雷州半岛人工增雨试验基地等重大项目，开展南渡河调水工程前期工作；加快建立用水商品化机制，大力推广节水灌溉技术和建设节水灌溉设施，在更大范围有效解决干旱问题。认真抓好省人大议案农田水利和现代标准农田项目建设。兴修800公里农村公路，行政村逐步开通客运班车。基本实现通行政村公路硬底化，自然村通路、通电、通电视、通电话。实施农村“信息搭桥”工程，年内完成所有行政村通宽带并建立信息化体验站，实现“网络到村、信息入户”。加大农村和革命老区扶贫工作力度，提高自我脱贫能力。动工建设乌石国家级中心渔港。加快建立农产品交易和农资供应市场长效管理监督机制，加强农产品收购价格监管，切实解决农产品流通环节克扣伤农问题，坚决维护农民利益。

　　扎实推进新农村建设。坚持中央新农村建设“二十字方针”和市委“六个一”要求，加强规划引导，推进乡村规划全覆盖，力争年内完成村庄规划达到自然村总数的40%以上；全面推行“政府主导、农民主体、干部服务、社会参与”的运行模式，以“四通五改六进村”为载体，尊重农民意愿，以经济为中心、产业为依托、生态为特色、旅游为纽带，实施集中连片规划建设，在全市推进35个生态文明区、500个各级生态文明村和100个卫生村建设，推动新农村建设从亮点到亮线、从亮线到亮片集群发展。

　　三、完善基础设施体系和功能，加快粤西城镇群中心建设

　　推进以交通为先导的重大基础设施建设。全年安排重点建设项目47个，其中续建、新建项目34个，前期预备项目13个，年度计划投资63亿元。一是着眼打造区域性国际航运中心，推进港口重大项目建设，提升大西南出海主通道地位。建成湛江港30万吨级航道和徐闻海安新港码头一期工程；推进宝满港区集装箱码头、霞山港区成品油码头、调顺岛港区7万吨级航道和东海岛港区建设，完善服务设施体系，提升港口功能；实施县域港区规划，加快县域港口建设。二是着眼大工业、大港口的衔接配套，完善重大功能性基础设施体系。动工建设茂湛铁路、湛徐高速公路、东海岛跨海大桥、南三大桥和鉴江供水枢纽工程，继续推进电力配套设施建设，完善供电网络；实施疏港公路二期、海湾大桥西连接线和325国道绕市区段建设工程；积极推动广湛高速公路官渡出入口立交项目建设。

　　推进以湛江市区为中心的城镇群建设。按照省第十次党代会为湛江的发展定位，加快建设以市区为龙头，县（市）城区和中心镇、专业镇为主轴，建制镇和国营农场场部为基础的粤西城镇群中心。一是强化城乡规划。贯彻实施《城乡规划法》，规范规划修编和实施程序。修订完善《湛江市城乡规划管理办法》和《湛江市城市规划管理技术规定》。编制麻章区分区规划和东海岛等控制性详细规划以及城市综合交通等一批专项规划。健全城市规划实施机制，严格按照“公开、公正、公平”原则，坚持规划委员会集体决策审批，确保规划审批的严肃性；加强规划审批和实施全过程、全方位监督管理，坚决依法严肃处理违法建设行为并公开曝光。二是加快中心城市建设。进一步完善城市道路体系，重点改造建设南方路网、奋勇大道、新湖大道、体育南路、体育北路、北站路和市区出入口路段。推进海滨大道扩建工程，强力推动城中村、城郊村和旧城区改造，精心设计和建设一批特色建筑、创意街景，形成一批精品片区，塑造特色城市景观，全面提升城市形象。坚持公交优先发展，优化公交线路网络，增建换乘站、候车亭，加快麻章、海田、汽车南站公交主枢纽站场改造。三是推进县（市）城区建设。充分发挥县（市）城区集聚生产要素、服务经济产业的作用，加快建设工业集中区和居住集中区，引导工业、服务业、房地产业集聚，促进产业与城区的对接融合，以产业集聚加快城区发展，以城区发展带动产业升级，促进发达地区产业和本地农民向县城转移，使城区的成长过程成为城乡共同繁荣、一体化发展的过程，成为县域经济发展的龙头。四是坚持优先发展中心镇。全面落实省、市加快中心镇建设的各项政策措施，按照“统一规划、突出特色、配套建设、综合开发”的原则，加快公共服务设施建设，大力发展二、三产业尤其是服务业，壮大中心镇经济实力，提高对农村劳动力的吸纳能力，增强对广大农村的辐射带动作用。

　　推进以优化发展环境为目标的城镇管理体制建设。完善“两级政府、三级管理、四级网络”的城市管理体制，按照重心下移、权责一致的原则，构建“统一领导、分级负责、条块结合、依法管理、社会监督”的城市管理格局，赋予街道、社区更多的管理权限，发动广大群众积极参与城市管理。推行城镇路段分类管理，开展评选示范街、样板路和卫生街区活动，合理设置临时摆卖街区和农副产品定时批发交易市场，实现城镇管理从突击粗放向规范精细转变。以市场化为取向，推动城镇道路保洁、绿化养护、污水和垃圾处理等管理和运营机制创新。推进数字化城市管理，完善城镇管理热线投诉系统，提高城镇管理效能。

　　四、着力推进改革开放，进一步增强发展动力和活力

　　深入推进重点领域改革发展。毫不动摇地坚持社会主义市场经济改革方向，为科学发展提供体制保障和强大动力。一是继续深化国有企业改革。完善企业法人治理结构和业绩考核体系，强化国有资本收益收缴和预算管理。支持国有企业引入战略投资者，加快产权多元化进程，优化经营机制，盘活存量资产，化解历史遗留问题和债务负担。支持湛江港集团、湛化集团等符合条件的企业上市融资，推动市商业银行实行股份制改革。有序推动40至50家劣势国有企业平稳退出市场，力争两年内基本完成市属劣势国有企业退市工作。二是继续完善公共财政管理体制。进一步调整和优化财政支出结构，把财政支出重点投向公共服务和民生领域。加强财政专项资金监管，财政资金扶持项目符合条件的实行公开招投标制。扩大财政支出绩效评价范围，建立财政资金跟踪问效机制。全面推行市级行政单位财务核算集中监管、县级国库集中支付以及村财镇管。加强税收征管和非税收入电子化征管。落实省对县（市）、市对区激励型财政政策，充分调动各县（市、区）开拓财源、发展经济的积极性。三是加快事业单位改革。支持经营开发服务类事业单位转企改制，全面推进社会公益类事业单位用人制度和分配制度改革，理顺行政支持类事业单位管理体制。推进华侨管理区体制改革。四是促进民营经济大提高大发展。全面落实促进民营经济发展的政策措施，重点解决市场准入、融资、社会服务等问题，支持民营资本依法进入基础设施、基础产业、公用事业等行业，推动民营经济与公有制经济、外资经济融合互动，发展股份制经济。大力扶持一批创新能力强、经营机制活、发展后劲足的民营企业做大规模、增强实力。

　　努力扩大外贸出口。加快转变外贸增长方式，优化出口商品结构，扩大技术含量高、附加值高的产品出口，提高传统出口商品质量，推动加工贸易转型升级，大力发展服务贸易。积极培育多元化和自主品牌外贸经营主体，支持更多企业取得进出口经营权，熟悉国际通行规则，争取出口产品国际认证，积极应对技术壁垒和反倾销，努力在国际市场上谋取更大发展空间。完善口岸设施，推进大通关建设。重点培育一批年出口规模超过3000万美元的外贸企业和年出口额超1亿美元的县（市、区）。

　　积极有效利用外资。充分发挥优势，重点引入战略投资者，引导外资更多地投向重化工业、农海产品加工业、现代农业和现代服务业等领域；坚持对新引进项目进行综合评价，推动招商引资向招商选资转变，优化利用外资结构，提高利用外资质量。创新招商引资形式、方法和手段，精心策划、包装、推介招商项目，积极做好引进项目的跟踪服务工作，提高招商引资实效。进一步调动各方面招商引资的积极性，促进实际利用外资不断增长。

　　加强和促进区域合作。创新区域合作的机制和方式，主动参与中国—东盟自由贸易区、环北部湾和大西南地区、港澳地区、珠三角的经济技术合作，扩大发展空间。大力推动与21个国内友好城市在港口、产业、市场、旅游、环保等方面的合作，促进资源、资金、技术、品牌等要素共享，形成优势互补、良性互动、共同繁荣的友城发展新格局。切实提高与澳大利亚凯恩斯市、俄罗斯谢尔普霍夫市等国外友好城市的合作水平。协助省办好第五届珠三角地区与山区及东西两翼经济技术合作洽谈会，进一步扩大区域经贸交流与合作。

　　五、强化资源能源节约和环境保护，推进生态文明建设

　　提高土地利用效率和集约化程度。充分发挥规划对土地开发利用的统筹和综合调控作用，严格执行土地利用总体规划和年度计划，实施城镇建设用地规划、征用、开发、管理“四统一”，进一步健全科学的土地收购储备和出让制度，完善和规范农村土地租赁和留用地进入市场交易监督机制，严格控制城镇私人住宅建设。加强基本农田保护区管理，完成土地整理10万亩。开展第二次土地调查。合理调整用地结构、布局和开发时序，市、县两级政府高度垄断土地一级市场，大力盘活土地二级市场，进一步优化建设用地配置和供给。制定实施《湛江市投资项目准入制度》，对新引进项目的土地利用率、建筑容积率等进行综合评价，确保土地供应向产业带动能力强、产出效益好、资源能源消耗少的项目倾斜，提高工业用地的使用效益。建立已审批土地开发使用掌控机制，督促各类开发区、各类企业搞好存量土地的挖潜增效，加大非农建设闲置土地的回收处置力度，促进土地集约高效利用。

　　着力抓好节能降耗和发展循环经济。全面落实各级政府和企业节能降耗工作责任制，建立单位GDP能耗统计、监测和考核体系。着重抓好电力、石化、建材、纺织印染、造纸、制糖等重点行业和100家重点耗能企业的节能考核，实行重点企业能源审计和能源利用状况公告制度。推进建筑节能节材，大力开发和推广应用太阳能、风能等新型能源以及环保轻质新型材料。完善公共建筑、生活小区中水回用设施，创建节水型城市。严格执行对高耗能、高污染工艺、设备及生产能力限期淘汰制度，对不按期淘汰的企业，依法予以关停。全面推行清洁生产，组织实施44个风力、压差发电和水资源再生利用等循环经济示范项目，培育一批具有较高资源利用率、较低污染排放率的循环经济示范企业和示范园区。年内确保完成国家和省下达的节能降耗约束性指标任务，实现工业重复用水率70%以上，工业固体废弃物综合利用率90%以上。

　　加强生态环境保护和建设。制定《湛江市生态文明建设规划》，明确建设城乡协调、生态文明的科学发展试点市的近、中、远期目标和任务。完成第一次全国污染源普查任务。实行环保一票否决制度，严格建设项目环境准入管理，对于不符合城市总体规划、不符合产业政策、不符合环保要求的项目，坚决不予审批，从源头上控制污染源。坚持以保护饮用水源为重点，加强九洲江、鉴江和南渡河流域的环境监测与整治，有效解决50万人饮水安全问题。加大南桥河、赤坎江二期等市区河流、城市水体排泄截污及湛江港湾养殖综合治理力度，严厉打击违法排污行为。加强餐饮业油烟和机动车辆废气排放污染治理。大力实施农村清洁能源利用工程，推广户用沼气，减少农村面源污染。建成赤坎污水处理厂二期工程，动工建设麻章、坡头污水处理厂，积极推动县（市）城区加快建设污水处理厂和生活垃圾处理场。合理调整污水、垃圾处理收费，促进城市环保事业发展。实施公共交通清洁能源改造，年内在市区推出100辆液化天然气公交车运营。深入实施城乡清洁工程，巩固和提高已有成效，加快建立完善长效管理机制，全面持久净化、美化、绿化城乡环境。建成南国热带花园，继续实施东坡荔园、三岭山森林公园改造，建设海湾大桥桥东公园、滨湖公园一期工程和省级湖光红树林湿地公园，新建一批小游园、小绿地，续建金沙湾观海长廊三期工程，市区海滩涂种植红树林1000亩，年内增加城市绿地100万平方米以上。开展创建国家卫生城市、国家环保模范城市和国家绿化模范城市申报工作。继续推进创建国家生态园林城市和国家级生态示范区、林业生态县、省级生态示范镇以及绿色社区等活动，支持坡头区、吴川市创建林业生态县。全市新增造林面积24万亩。统筹解决人口问题，努力完成人口控制目标，减轻环境压力，实现人口与资源环境相协调。

　　六、全面推进社会建设，增强社会创造活力

　　大力提高社会文明程度。切实把社会主义核心价值体系融入精神文明建设全过程，抓好爱国主义、集体主义和社会主义思想教育，深入开展以“八荣八耻”为主要内容的社会主义荣辱观和现代公民教育活动，形成知荣辱、讲正气、促和谐的良好社会风尚。加强科普和生态文明教育，倡导科学文明健康的生活方式。继续完善基层民主制度，保障人民群众的知情权、参与权、表达权和监督权。完成村委会、社区居委会换届选举工作。广泛开展文明城市、文明行业等各类精神文明创建活动，进一步提高广大群众的现代文明素质。推进“五五”普法活动，深入开展法治教育，增强法治观念，弘扬法治精神，形成自觉依法行使权利、履行义务的社会氛围。

　　大力发展教育事业。完善义务教育经费保障机制，巩固农村免费义务教育，从今年春季学期起免除城镇义务教育阶段学生学杂费和课本费，实现全市城乡免费义务教育。健全学生资助制度，保障经济困难家庭、进城务工人员子女平等接受义务教育。建立农村义务教育校舍维修长效机制。加快普及高中阶段教育，增加高中教育学位，力争年内高中阶段教育毛入学率达到65%。加快发展职业技术教育，建设一批重点职业院校、重点专业和实训中心，培养适应经济社会发展需要的技能型人才和实用人才。支持在湛高校扩大办学规模，推动广东医学院坡头新校区建设工作。鼓励和规范发展民办教育，重视学前教育，关心特殊教育。

　　大力发展科技事业。加强科研基础条件建设，加快实施广东省海洋开发研究院起步项目。鼓励和支持驻湛高校建立重点实验室、重点学科和科研基地，开发高新技术和实用技术，更好地为湛江经济社会发展服务。推动企业与高校、研究所联合创办研发机构，加强产学研协作，增强自主创新能力。年内争取建立省级特色产业基地1个，市级专业镇4个、工程中心和农业技术创新中心3个、企业技术研发机构3－5家。加大知识产权创造、运用、管理和保护力度，全年专利申请量和授权量分别增长10%以上。继续加强人才引进、开发、培训工作。

　　大力发展文化事业和文化产业。推进全省东西两翼文化建设工程，有效整合文化资源，努力培育地方特色文化品牌。加强非物质文化遗产、历史文化资源、民间艺术保护和传承，启动全国第三次文物普查，完成雷州石狗保护工程。积极发展文学艺术、新闻出版和广播影视业。深入推动乡村、社区、企业、校园等文化建设，因地制宜开展特色文化街区和文化村、文化室、文化节活动，丰富城乡群众精神文化生活。完善公共文化基础设施，建设市文化艺术中心，加快市区数字电视整体转换。举办市第七届艺术节。充分利用我市被列为全省文化体制改革试点城市的契机，加快文化体制改革，发展一批具有较强实力和竞争力的文化市场主体，促进文化事业和文化产业大发展大繁荣。

　　大力发展公共卫生和体育事业。建设覆盖城乡居民的公共卫生服务体系、医疗服务体系、医疗保障体系、药品供应保障体系，全面推广“平价医院、平价诊室、平价病房”活动，推进中医强市建设，为群众提供安全、有效、方便、价廉的医疗卫生服务，推动全国药品价格改革试点市工作，努力解决群众看病就医问题。继续改造乡镇薄弱卫生院，推进乡镇卫生院信息化和农村卫生站规范化建设，广泛开展卫生镇（村）、卫生街区和初级卫生保健达标创建活动。继续推进城镇居民医疗保险国家试点城市工作，今年参保新增25.8万人，参保率达到80%以上。完善新型农村合作医疗制度，年内实现农村人口参合率90%以上。加快体育场馆建设，积极做好湛江籍运动员参加2008年北京奥运会的各项工作，举办市第九届体育节、第二届国际龙舟邀请赛、市职工运动会、市第三届社区运动会等赛事活动。

　　大力推进各项社会工作。深入开展“双拥”活动，做好国防动员、民兵预备役和人民防空工作，加强基层武装部建设，促进军政军民团结。抓好民族宗教工作，全面落实侨务政策，依法保护归侨、侨眷合法权益。完善物价管理、监测、预警机制。加快档案信息化建设。巩固殡葬改革成果。实施地名公共服务工程。扎实做好统计、外事、对台、地震、修志以及妇女儿童、老龄等工作，进一步促进社会事业协调发展。

　　七、着力保障和改善民生，共建共享和谐社会

　　促进社会充分就业。认真贯彻落实《就业促进法》，建立和完善公共就业服务体系，认真落实小额贷款、税费减免、社会保险补贴和设立创业扶持资金等多种措施，努力扩大就业。进一步办好创业示范基地，以创业带动就业。全面实施省“三年30万”城乡就业援助工程，开展“一帮一”扶助困难群体就业活动，确保新增“零就业家庭”半年内有一人就业。积极实施“农村青年技能培训”、“高技能人才培养”、“创业带动就业”和“退役士兵职业技能培训”工程，组织农村青年技能培训5万人，其中减免费技能培训2.5万人，转移农村劳动力就业20万人。城镇年内新增就业岗位4万个；下岗失业人员再就业6000人，其中“4050”人员就业2000人。继续扩大残疾人就业，确保年内就业率达到85％以上。

　　完善社会保障体系。着力巩固和完善企业退休人员基本养老金、企业职工最低工资、低收入家庭困难补助、失业人员失业保险、工伤保险和城乡居民最低生活标准等保障防线。健全优抚安置保障长效机制。全市企业基本养老保险增加3万人、失业保险增加2万人、工伤保险增加3万人、城镇职工医疗保险增加2万人。以被征地农民养老保险为突破口，在有条件的地方探索建立农村养老保险制度。着力做好农村贫困群众生活保障，年内增加农村低保2.1万人。提高城乡孤儿保障帮扶工作水平。

　　推进城乡安居工程。建立健全城镇住房保障制度，出台《湛江市经济适用住房管理办法》，加强廉租住房和经济适用住房建设，力争市及县（市）城区对人均住房建筑面积10平方米以下的城镇低保家庭基本做到应保尽保，并将保障范围扩大到低收入住房困难家庭。继续推进农村困难群众茅草房、泥砖房改造工程，年内完成2万户改造任务；建设水库移民新房5000户，解决2万名水库移民住房困难问题；新建村级敬老院120间，解决2500多名农村五保老人住房难。

　　着力建设平安湛江。完善政府统一领导的应急管理体系和应急工作机制，加强对自然灾害和气候变化的监测、预报、预警和防控，及时应对和处置突发公共事件。全面贯彻《广东省食品安全条例》，深入实施食品药品放心工程，动工建设国家海产品质量监督检验中心，完善农产品检测网络，继续深入开展产品质量与食品安全整治工作，实施生产、加工、消费全过程监控，确保人民群众用上放心的食品药品。建立价格调控目标责任制，强化市场价格监督和规范秩序，严厉打击各种价格违规违法行为，确保市场价格尤其居民生活必须品价格基本稳定。严格安全生产监管，加大安全隐患整治和重大责任事故处理力度，加强消防工作，有效防止和遏制重特大事故发生。健全信访工作责任制和矛盾纠纷排查调处机制，坚持领导干部基层大接访、政法系统干部基层大巡访和领导包案制度，妥善解决民生信访突出问题，从源头上减少不稳定因素；全面完成乡镇综治中心和行政村综治工作站建设，建立农村维稳律师团和手机信访信息系统。力争年内信访总量下降50%以上。大力推进公安“三基”工程和社会治安视频监控系统建设，完善社会治安防控体系，依法严厉打击各种违法犯罪行为，保持打击走私高压态势，严密防范敌对势力渗透破坏活动，保障人民群众安居乐业。

　　八、加强政府自身建设，努力提高行政能力

　　推进政府管理创新。加快政企分开、政社分开、政事分开、政资分开、政府与中介机构分开，更多地运用经济手段、法律手段调节经济活动，更好地为市场主体服务和创造良好环境。切实加强社会管理和公共服务，优化公共资源配置，加强公共设施建设，完善社会管理制度，提高社会管理水平，努力实现基本公共服务的均等化，促进社会公平正义。合理划分市与区、县（市）的事权和财权，明确不同层级政府、政府各部门及部门内部的职责权限，切实解决职责交叉、权责脱节问题。设立工作创新奖，鼓励各级机关和干部群众解放思想敢闯敢试，创造性工作。继续推行政务、厂务、村务和公共企事业单位办事公开，完善新闻发言人、政府网站、政务公示栏等公开渠道，全面改造市区政务公开电子触摸系统，健全政府信息公开发布、监督和评议制度，提高政府工作的透明度。

　　全面推进依法行政。严格执行《湛江市行政机关及公务员首问首办责任制实施办法》、《政府工作部门行政首长问责办法》、《行政效能过错责任追究办法》，保证行政机关及其工作人员按照法定权限和程序行使职权、履行职责。不断完善权责明确、行为规范、监督有效、保障有力的执法体制，做到有权必有责、用权受监督、侵权要赔偿、违法须追究。健全政府重大决策和重大事项专家咨询论证、社会公示听证和合法性审查等制度，促进政府决策科学化、民主化和制度化。完善规范性文件审查、公开发布制度和异议处理机制，加强行政复议工作，依法开展行政应诉。坚持实行经济责任审计结果公告制度，向社会公开整改情况接受监督。自觉接受人大及其常委会、政协、各民主党派和人民团体、人民群众和新闻舆论监督，积极办好人大代表议案、建议和政协提案。

　　深入开展机关效能建设活动。以落实部门服务公开承诺为重点，不断把机关效能建设活动引向深入，确保各级政府机关作风明显改进，服务能力明显增强，办事效率明显提高。继续推进行政审批制度改革，精简和调整不适应市场经济要求的审批事项和收费项目。清理各类与行政审批相关联的中介服务事项，提高政府公共服务水平。深化电子政务建设，充分发挥电子行政审批系统和电子监察系统的作用，不断提高行政审批质量和效率。强化行政服务中心“一站式”服务功能，全面推行首席代表制和告知承诺、并联审批、限时办结制度，提高窗口现场办结率。推进新行政服务中心建设。加强行政效能监察，严肃查处各种行政不作为、乱作为和效能低下行为。

　　大力加强廉政建设。深入贯彻《湛江市建立健全教育、制度、监督并重的惩治和预防腐败体系实施办法》，全面落实党风廉政建设责任制，认真履行政府廉政承诺，严格执行“十项纪律”等廉洁自律规定，树立清正廉洁的政府形象。进一步完善建设工程招投标、土地出让、产权交易、政府采购等制度，加强对重要部门、重要岗位、关键环节权力行使的监督。加大治理商业贿赂工作力度，规范公务接待，降低行政成本。严肃查处领导机关和领导干部以权谋私等违法违纪行为，切实纠正损害群众利益的不正之风。进一步加强各级政府和机关思想作风、领导作风、工作作风建设，力戒官僚主义、形式主义，大力改进文风会风。加强公务员教育和培训，弘扬艰苦奋斗精神，厉行勤俭节约，反对铺张浪费，努力建设为民务实清廉的公务员队伍。

　　各位代表！面对新时期新使命，我市发展站在新的历史起点上。建设粤西城镇群中心，是湛江现代化进程中的重要战略目标；建设城乡协调、生态文明的科学发展试点市，是湛江增创新优势、实现新跨越的必由之路。我们的事业任重而道远，责任重大而光荣。让我们在市委的正确领导下，全面贯彻党的十七大精神，深入贯彻落实科学发展观，解放思想，同心同德，开拓创新，奋发图强，为湛江经济社会全面振兴、全市人民富裕安康而努力奋斗！
